# Some Effects of Aging on the Surface Area of Portland Cement Paste

**DOI:** 10.6028/jres.064A.016

**Published:** 1960-04-01

**Authors:** C. M. Hunt, L. A. Tomes, R. L. Blaine

## Abstract

A hardened cement paste cured at room temperature, from which part of the evaporable water has been removed by vacuum drying, has been studied. The surface area has been shown to decrease with time depending upon the amount of evaporable water left in the paste. This change is the opposite of that usually observed during hydration and probably represents some collodial growth phenomena analogous to aging observed in other collodial gels. Both water vapor and nitrogen adsorption measurements have been used to show the effects of aging in cement paste.

Wet or dry paste is shown to undergo less change than paste of intermediate evaporable water content, so that if surface area after storage is plotted as a function of evaporable water content, a curve with a minimum is obtained. With increasing storage temperature there is some indication that this minimum might shift towards lower water content.

Aging is shown to occur during the initial drying of a cement paste, so that even the initial surface area of a cement paste depends upon the manner in which the paste has been dried.

## 1. Introduction

Aging phenomena have been observed in many colloidal solids. Colloidal systems are unstable and with time there are usually chemical and physical changes transforming the system towards a more stable state. The gel produced by the hydration of Portland cement is also subject to such processes, and this fact is undoubtedly of some importance in such properties of concrete as creep, drying shrinkage, and the development or loss of strength. However, comparatively little attention has been given to aging phenomena per se.

One of the changes usually associated with aging is a decrease in surface area [1, 2, 3, 4].^2^ It is difficult to demonstrate such a decrease in cement paste, because as long as hydration continues, new gel particles are formed and surface area increases [5, 6]. However, if part of the water is removed, the hydration process is disturbed, and it is possible to observe other effects. The interruption of hydration can occur in very old specimens by self-desiccation [7, 8], or it may be brought about in younger specimens by drying. The present paper considers changes in surface area of a hardened cement paste from which enough water has been removed to stop, or at least seriously curtail hydration. Such changes are considered important, partly because they affect the reproducibility of the measurements, but they are perhaps even more important in the interpretation of vapor sorption and surface area measurements.

## 2. Methods and Materials

### 2.1. Preparation of Cement Pastes

Pastes were prepared from cement, meeting the ASTM requirements for type I cement. Three different cements were used during the course of these experiments. Their composition is given in table 1.

To minimize contact with air dining mixing, the pastes were mixed in a soil density balloon.^3^ A water-cement ratio of 0.5 was used. Specimens were prepared in the form of cylinders 1/2-in. long and 1/2-in. in diam. The initial hydration of the paste, referred to here as curing, took place in the molds for the first 24 hr and thereafter in paraffin sealed quart jars. A high relative humidity was maintained by 20 to 30 ml of water in the bottom of each jar. Curing time was 1 month except where otherwise designated.

### 2.2. Storage of Pastes

The cured pastes were crushed, divided into a number of portions, and vacuum dried in the apparatus used in the determination of nonevaporable water. The drying period of the different portions ranged from as short as 1 hr to as long as 1 week. They were dried for different periods of time in order to obtain specimens differing in evaporable water content. The partially dried pastes were stored in sealed glass tubes. Dunmore gage elements were present in some of the tubes to permit measurement of relative humidity during storage. Storage times of 1 month, 3 months, and 21 months were investigated. Specimens were stored at 21° C except in one experiment in which they were stored 1 month at 37.8° C (100° F) and 105°C.

### 2.3. Determination of Evaporable and Nonevaporable Water

The room temperature vacuum drying procedure used in the determination of evaporable and nonevaporable water was similar to that of Copeland and Hayes [9]. However, since the stored specimens received partial drying prior to storage, they were subsequently dried during this procedure so that each specimen ultimately received a total of 2 weeks of drying, including drying prior to storage. Poststorage drying times were as short as 1 week or as long as 2 weeks, depending on the prestorage drying treatment of the particular sample of paste. Evaporable water was the loss of weight during vacuum drying after storage. Nonevaporable water, *w_n_*, was the subsequent loss of weight during ignition to constant weight at 1,050° C after correction for carbon dioxide in the specimen.

### 2.4. Measurement of Water Vapor and Nitrogen Adsorption

Water vapor adsorption measurements were made after the removal of evaporable water from the paste. The measurements were made in an apparatus which has been previously described [10] and which uses a stream of nitrogen at atmospheric pressure to transport the water vapor. Values of *v_m_* were calculated by the BET equation [11], where *p* is the pressure of water vapor in equilibrium with the specimen, *p*_0_ is the vapor pressure of pure water, *C* is a constant related to the

$$\frac{p}{v\left(p_{0}-p\right)}=\frac{1}{v_{m}C}+\frac{\left(C-1\right)p}{v_{m}C\;p_{0}}$$ (1)

heat of adsorption, *v* is the amount of water adsorbed per gram of adsorbent, *v_m_* is the amount of water required for monolayer coverage of the adsorbent.

Nitrogen adsorption measurements were made with an apparatus similar to that described by Emmett [12]. The vacuum dried specimens were further outgassed overnight at 100° C before measuring their nitrogen adsorption. BET surface areas were calculated, assuming each nitrogen molecule to cover an area of 16.2A^2^.

## 3. Results

### 3.1. Changes in Paste Stored at Room Temperature

**Figure 1:**
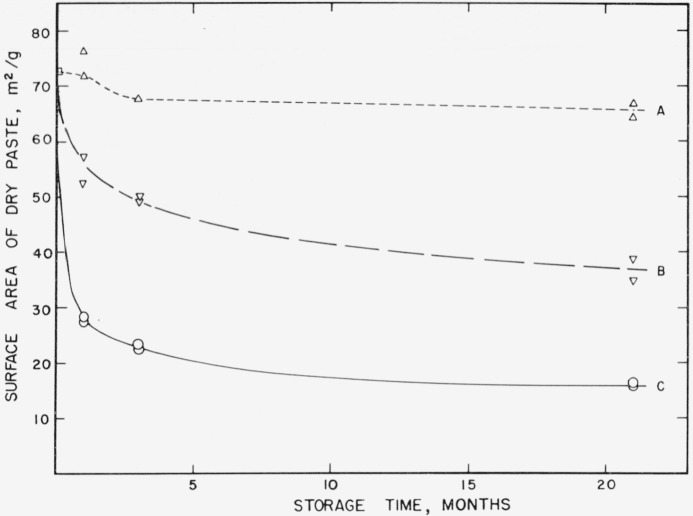
Changes in nitrogen surface area of a hardened cement paste during storage at 21° C. A–––Paste containing 1% evaporable water B— —Paste containing 17 to 20% evaporable water C___Paste containing 10% evaporable water Specimens prepared from cement No. 5.

The surface area of a hydrated cement paste was measured before and after storage, and large decreases were observed. This is illustrated in figure 1 where nitrogen areas of specimens which contained different amounts of evaporable water during storage are plotted as a function of storage time. The rate and amount of decrease in surface was profoundly affected by the evaporable water content of the paste. The specimens containing 10 to 20 percent evaporable water changed rapidly during the first month and more slowly thereafter. Specimens containing approximately 1 percent evaporable water changed very slowly throughout the 21 months of storage.

Since free water plays such an important role in the aging of hardened cement paste, it is necessary to consider its effect more fully. In figure 2a nitrogen surface areas of dried pastes are plotted as a function of the evaporable water content, as determined at the end of storage. The horizontal line, *A*, represents the average surface of unstored paste after 2 weeks of uninterrupted drying. It is clear from the figure that there is an “optimum” evaporable water content during storage at which the greatest decrease in surface area occurs. The “optimum” in figure 2a appears at about 10 percent evaporable water. This roughly corresponds to the amount of water required to fill the gel pores, of this particular paste as estimated by the method of Copeland and Hayes [13].

In figure 2b the same surface areas have been plotted as a function of the relative humidity in the storage tubes. The maximum decrease took place at relative humidities between 40 and 60 percent. The relative humidity increased slightly with time except in tubes containing some of the driest specimens.

Water vapor adsorption also shows the effects of storage. In figure 2c, *v_m_/w_n_* ratios have been plotted as a function of evaporable water content. A constant *v_m_/w_n_* ratio implies that the same amount of gel always requires the same amount of evaporable water for monolayer coverage. Pastes prepared from the same cement usually have the same *v_m_/w_n_* ratio regardless of curing time or water-cement ratio [5]. However the results in figure 2c show that measurable decrease in *v_m_/w_n_* ratio has taken place during storage. The decrease is related to the amount of evaporable water in the specimen during storage as in the case of nitrogen adsorption.

In figure 2d nonevaporable water content is plotted as a function of evaporable water content.

The increase in nonevaporable water during storage is seen to be negligible except for possibly one paste which was stored 21 months and contained 16 percent evaporable water at the end of storage, indicating that the changes represented in figures 2a, 2b, and 2c are due to modification in the colloidal structure of the hydration products already present.

### 3.2. The Effect of Temperature on Storage Changes

Samples of paste containing different amounts of evaporable water were also stored 1 month at 37.8° C (100° F) and 105° C. The changes in nitrogen surface area and *v_m_/w_n_* ratio are shown in figure 3a and 3b, respectively. The corresponding curves at 21° C (from figs. 2a and 2c) are replotted in the figures for comparison. The results suggest that the minimum in the curve may be displaced to lower evaporable water contents as the temperature is raised. From figure 3a it may be seen that the decrease in nitrogen surface area during storage became less with increase in temperature for the more humid specimens, but for dryer specimens precisely the opposite behavior was observed. This statement also seems to apply to *v_m_/w_n_* ratios shown in figure 3b, when 21° and 37.8° C storage are compared, but at 105° C the decrease in *v_m_/w_n_* ratio during storage was greater for both the humid and dry specimens.

There was slightly more evidence of continued hydration during storage at the higher temperatures than at 21° C. At 105° C an increase in nonevaporable water content of 0.8 percent was observed in the most humid specimen during 1 month of storage, while nonevaporable water in the driest paste decreased 0.7 percent during this time. The corresponding changes in surface area and *v_m_/w_n_* ratio at 37.8° C were of the order of 0.2 to 0.3 percent. The changes at higher temperatures were therefore accompanied by small changes in the total amount of hydration products present.

### 3.3. Storage Changes in Younger Pastes

Figure 4a shows the changes in nitrogen surface area which were found to occur in pastes which were cured for 1 day and 7 days respectively and stored for 3 months. Decreases, depending on evaporable water content, are shown for both groups of specimens. However considerable hydration took place during storage, especially in the 1-day specimens, as evidenced by the significant increases in nonevaporable water shown in figure 4b. It is difficult to separate the effects of continued hydration from changes in hydration products already present in younger pastes.

### 3.4. Effect of Drying Rate on Nitrogen Surface Area

The foregoing results showing time-dependent changes in cement pastes also have important implications with respect to the determination of surface area itself. Since, as is shown in figures 1 and 2a, most of the decrease in surface area took place during the first month of storage, it is reasonable to suppose that significant decrease must also have taken place during the first week. If this is true, aging must also take place during the initial drying, and the measured surface area of a cement paste should depend upon the rate at which the specimen is dried.

**Figure 5:**
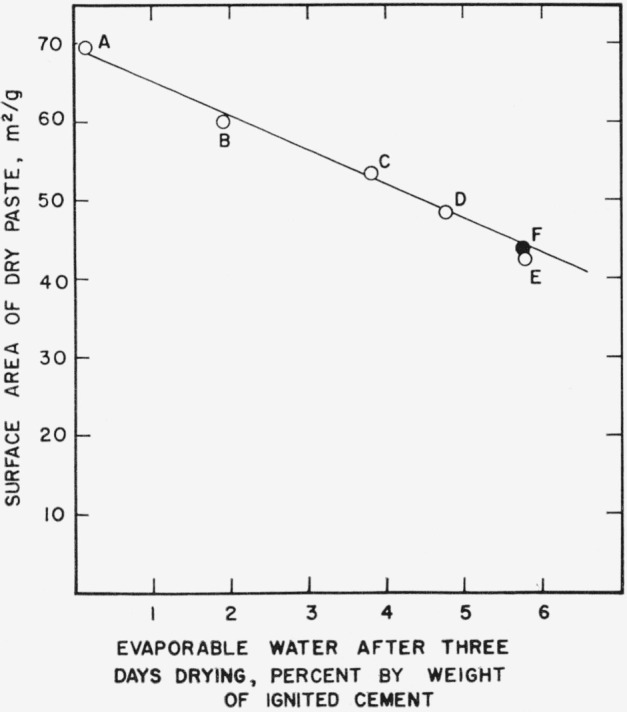
Nitrogen surface area of a hardened cement paste after drying at different rates. A—Crushed paste subjected to 1 week of vacuum drying at unrestricted rate B, C, D, E—Crushed paste subjected to 1 week vacuum drying with drying rate restricted by glass capillaries of different sizes. F—½-in. cylinder of paste subjected to 1 week of vacuum drying at unrestricted rate. Specimens prepared from cement No. 3.

Figure 5 shows the surface areas of six specimens of 1-month cement paste which were vacuum dried at different rates for 7 days. Evaporable water content after the first 3 days drying has been selected as a measure of drying rate. A specimen containing more evaporable water after this arbitrary period of drying passed through the critical stages of drying suggested by figure 2a more slowly than a specimen containing less evaporable water. As may be seen in figure 5, it has been possible to produce a variation in surface area of nearly 30 m^2^/g by merely restricting the rate of drying.

Five of the specimens represented in the figure were in the form of crushed paste while one was in the form of a ½ in. cylinder. A cylinder would dry more slowly due to restriction of diffusion by the paste itself. The largest surface area was obtained after a week of unrestricted vacuum drying of a crushed paste, a procedure similar to that of Copeland and Hayes [9]. This value is considerably greater than that obtained with the cylinder. However, when the rate of drying of crushed paste was restricted by sealing it in a closed tube with a tiny exit capillary, surface areas comparable with that of an uncrushed specimen could be obtained.

## 4. Discussion

The colloidal structure of hardened cement paste cured at ordinary temperature can undergo changes leading to a decrease in surface area which in some respects resemble aging effects observed in other colloidal gels. In a mature paste (1 month or older) where the rate of hydration is small, it has been shown that decrease in surface area is not accompanied by appreciable change in nonevaporable water content and is therefore due to some time-dependent modification of the gel already present. The effects of aging are reflected in both nitrogen and water vapor adsorption measurements, but changes in nitrogen adsorption capacity are relatively greater.

Powers and Brownyard [14] sometimes observed that cement pastes which had been dried and then exposed to water vapor, first gained and then lost weight. Powers [15] has also noted decreases in water vapor surface area of pastes stored at relative humidities between dryness and complete saturation and suggested some connection between this decrease and the anomalous behavior of dried pastes in water vapor. It seems very probable that the tendency of dry pastes to gain and then lose weight in water vapor is another manifestation of aging.

The greatest amount of change occurs in pastes from which part of the evaporable water has been removed. This observation suggests the possibility of some relationship between creep in concrete and the colloidal changes detected by surface area measurements. It has been shown that partially dried concrete specimens undergo greater creep (inelastic deformation under load) than saturated [16, 17] or oven-dried [17] specimens. A further qualitative similarity between aging changes and creep is that both become slower with time. Colloidal growth phenomena may afford a partial answer to the question why creep does not continue indefinitely at the initial rate.

At high temperatures aging processes usually take place more rapidly. Digestion on a steam bath is a familiar procedure for treating precipitates before filtration. However with cement pastes stored at 105° C it is also necessary to remove most of the evaporable water to achieve minimum surface area. If aging is promoted by the increases in reaction rates, and diffusion rates which accompany rise in temperature, perhaps thermal expansion tends to offset these factors by making it more difficult for primary particles to join together and form larger particles. Removal of most of the evaporable water would be expected to reduce thermal expansion and facilitate coalescence of colloidal particles. Although this interpretation of the mechanism of aging and the effect of temperature is speculative, Meyers [18] has shown experimentally that the apparent thermal expansion of hardened cement pastes exhibits a maximum depending upon the relative humidity at which the specimens were equilibrated before test, which in turn is a function of evaporable water content.

Not only do decreases in surface area, take place during storage, but the surface area itself is dependent upon the rate of drying. This observation helps to explain the fact that larger surface areas are obtained when specimens are crushed before drying than if they are crushed after drying. Either procedure can be made reproducible, and once the specimens are dried nitrogen surface areas are quite repeatable. The effect is analogous to obtaining different surface areas when specimens of different size are dried. Such behavior is reminiscent of results obtained in drying-shrinkage experiments in which specimens of small cross-sectional area have been found to shrink more than specimens of large cross-sectional area [19, 20, 21]. However, before attempting to interpret drying shrinkage results in the light of the present experiments, it should be pointed out that the slowest drying specimens in figure 5 probably dried much faster than the fastest drying specimen in the usual drying-shrinkage experiment. Nevertheless the study of colloidal aging effects should also cast some valuable sidelights on the mechanism of drying shrinkage.

## Figures and Tables

**Figure 2a:**
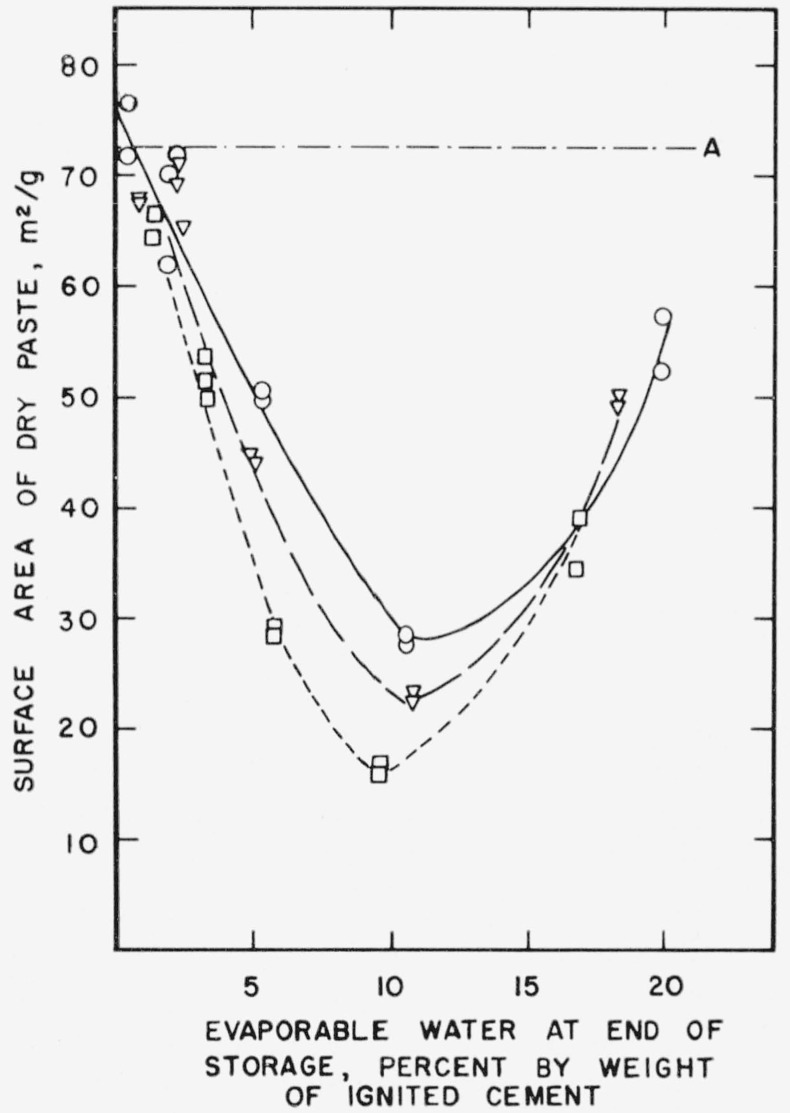
Changes in the nitrogen surface area of a hardened cement paste during storage as a function of evaporable water content. A–.–.–Original paste ○___Paste after 1 month storage at 21° C ∇ – – –Paste after 3 month storage at 21° C □-----Paste after 21 month storage at 21° C Specimens prepared from cement No. 5.

**Figure 2b:**
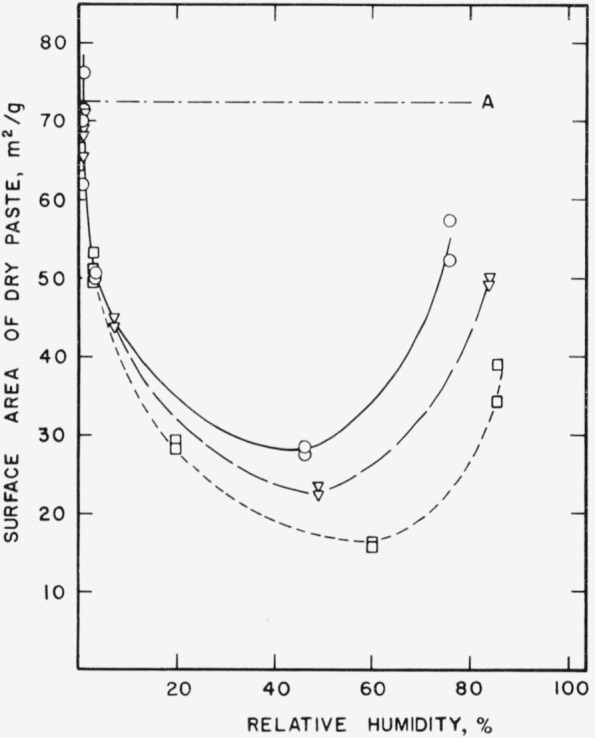
Changes in the nitrogen surface area of a hardened cement paste as a function of relative humidity. A–.–.–Original paste ○___Paste after 1 month storage at 21° C ∇– – –Paste after 3 month storage at 21° C □-----Paste after 21 month storage at 21° C Specimens prepared from cement No. 5.

**Figure 2c:**
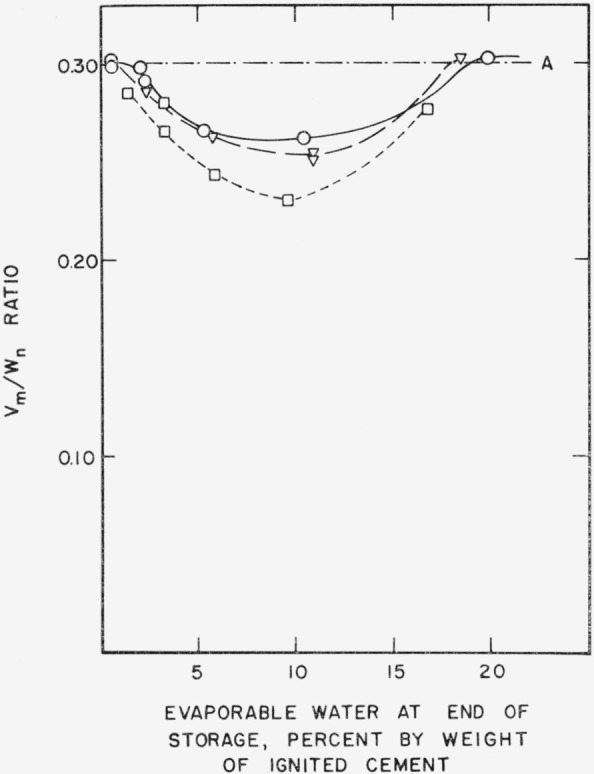
Changes in *v_m_/w_n_* ratio of a hardened cement paste during storage. A–.–.–Original paste ○___Paste after 1 month storage at 21° C ∇– – –Paste after 3 month storage at 21° C □-----Paste after 21 month storage at 21° C Specimens prepared from cement No. 5.

**Figure 2d:**
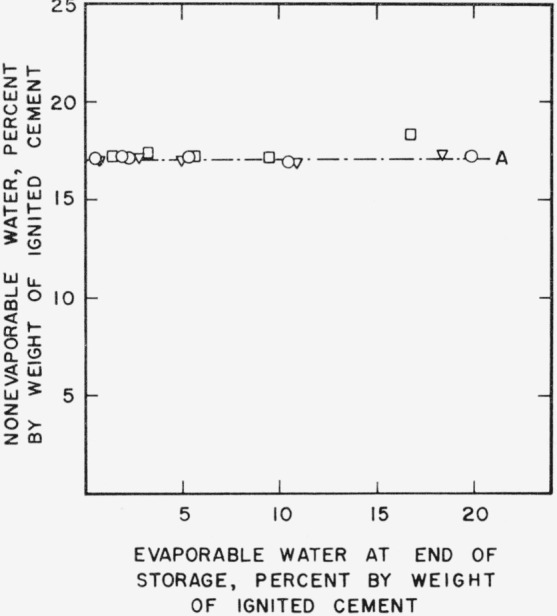
Changes in nonevaporable water content of a hardened cement paste during storage as a function of evaporable water content. A–.–.–Original paste ○    Paste after 1 month storage at 21° C Δ    Paste after 3 month storage at 21° C □    Paste after 21 month storage at 21° C Specimens prepared from cement No. 5.

**Figure 3a:**
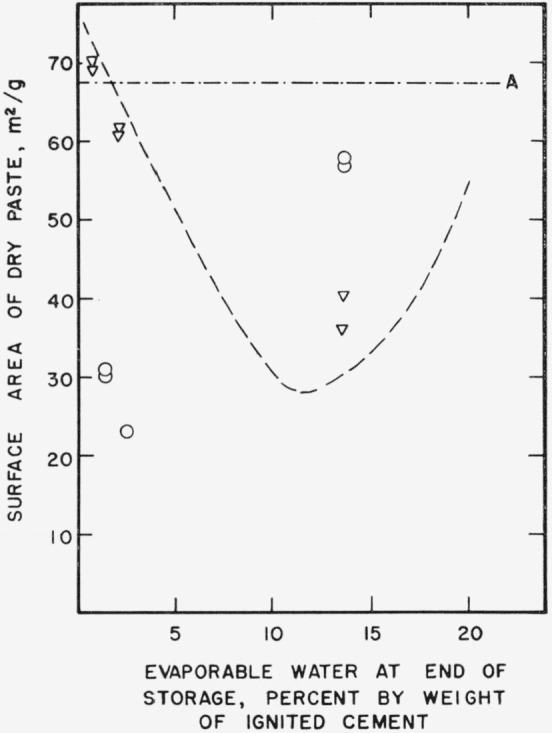
Changes in nitrogen surface area of a hardened cement paste stored at different temperatures. A–.–.–Original paste ∇    Paste after 1 month storage at 37.8° C (100° F) ○    Paste after 1 month storage at 105° C ------Paste after 1 month storage at 21° C (retraced from fig. 2a) Specimens prepared from cement No. 5.

**Figure 3b:**
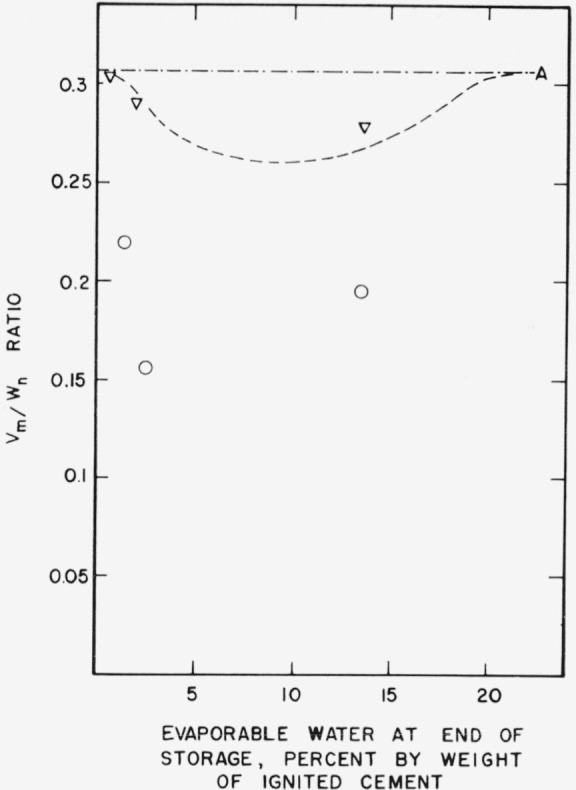
Changes in *v_m_/w_n_* ratio of a hardened cement paste stored at different temperatures A–.–.–Original paste ∇    Paste after 1 month storage at 37.8° C (100° F) ○    Paste after 1 month storage at 105° C -----Paste after 1 month storage at 21° C (retraced from fig. 2c) Specimens prepared from cement No. 5.

**Figure 4a:**
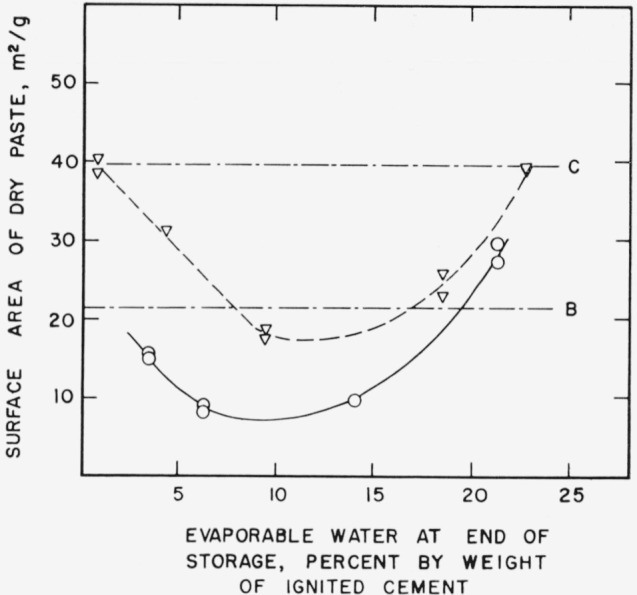
Changes in the nitrogen surface area of hardened cement pastes of different age as a function of evaporable water content. B–.–.–Original paste, cured 1 day ○___Paste cured 1 day, stored 3 months at 21° C C–.–.–Original paste, cured 1 week ∇-----Paste cured 1 week, stored 3 months at 21° C Specimens prepared from cement No. 1.

**Figure 4b:**
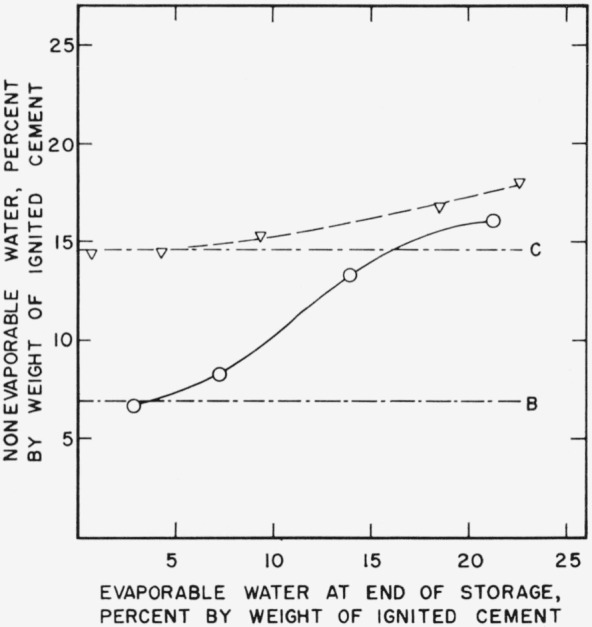
Changes in nonevaporable water content of hardened cement pastes of different age as a function of evaporable water content. B–.–.–Original paste, cured 1 day ○___Paste cured 1 day and stored 3 months at 21° C C–.–.–Original paste, cured 1 week ∇-----Paste cured 1 week, stored 3 months at 21° C Specimens prepared from cement No. 1.

**Table 1:** Chemical composition of cements

[Percentage by weight]			
	Cement		
	1	3	5^a^
			
CaO	64.0	64.1	62.8
SiO_2_	22.0	22.3	22.4
A1_2_O_3_	5.2	5.0	5.5
Fe_2_O_3_	4.6	3.0	2.8
MgO	1.6	1.9	4.2
SO_3_	1.5	2.0	1.9
Ignition loss	………	1.0	0.4
Insoluble residue	0.1	0.3	………

a Analysis performed on ignited paste.

## Footnotes

2 Figures in brackets indicate the literature references at the end of this paper.

3 Soil density balloons are supplied by the Rainhart Co., Austin, Texas. The type used in this work had a total volume of about 160 ml. The wall thickness was 0.4 to 0.5 mm, sufficiently strong to withstand the impacts of mixing.
